# Defining Critical Emergency Medicine (CrEM): A Delphi Study From Scandinavia

**DOI:** 10.1111/aas.70150

**Published:** 2025-11-24

**Authors:** Denise Bäckström, Miretta Tommila, Mette Pedersen, Thomas Lindner, Nanna Kruse, Robert Larsen

**Affiliations:** ^1^ Department of Biomedical and Clinical Sciences Linköping University Linköping Sweden; ^2^ Department of Leadership and Command & Control Swedish Defence University Karlstad Sweden; ^3^ Division of Perioperative Services, Intensive Care Medicine and Pain Management Turku University Hospital Turku Finland; ^4^ Department of Anaesthesiology and Intensive Care University of Turku Turku Finland; ^5^ Department of Anaesthesiology and Intensive Care Hospital of Lillebaelt Kolding Denmark; ^6^ Emergency Medical Services Region of Southern Denmark; ^7^ HEMS Den Regionale Akutlægehelikopter Denmark; ^8^ RAKOS Stavanger University Hospital Stavanger Norway; ^9^ University i Bergen K1 Bergen Norway; ^10^ Department of Anaesthesiology and Intensive Care Nordsjaellands Hospital Hillerød Denmark; ^11^ Emergency Medical Services Capital Region of Denmark Denmark; ^12^ Department of Anaesthesiology and Intensive Care Linköping University Hospital Linköping Sweden

## Abstract

**Background:**

Critical emergency medicine (CrEM) is one of four subspecialty pillars within anesthesiology and intensive care medicine, as defined by the Scandinavian Society of Anesthesiology and Intensive Care Medicine (SSAI). Despite its recognized clinical relevance, a comprehensive definition of CrEM has until now been lacking. The aim of this study was to establish a consensus‐based definition of CrEM and delineate its core components, competencies, and operational domains.

**Methods:**

A modified Delphi study was conducted among experts from the SSAI‐CrEM education program. The process involved two iterative rounds followed by external validation with alumni from previous CrEM programs. Statements for evaluation were generated from participant essays and refined by a steering committee of experienced consultants. Consensus was defined as ≥ 90% agreement.

**Results:**

Of 44 initial statements, 37 reached consensus and were organized into six thematic domains: (1) Core Function and Scope, (2) Competence and Training, (3) Work Environment and Challenges, (4) Interdisciplinary and Teamwork Approach, (5) Ethical and Decision‐Making Responsibilities, and (6) Need for Research and Continuous Development. CrEM was defined as a physician‐led, context‐adapted subspecialty focusing on rapid stabilization, life‐saving interventions, and high‐acuity care across diverse clinical, and prehospital environments. The results emphasize the need for structured training, ethical competence, leadership in multidisciplinary teams, and ongoing scientific development.

**Conclusion:**

CrEM constitutes a distinct and essential subspecialty within anesthesiology and intensive care medicine, bridging advanced emergency care across institutional boundaries. This study provides a structured definition and framework that may support curriculum development, clinical governance, and research initiatives within the field. Future work should aim to further validate these findings and guide the evolution of CrEM in both clinical and academic contexts.

**Editorial Comment:**

This Delphi process report presents the practice and training concepts for critical emergency medicine as a subspecialty of Anesthesia and Intensive Care Medicine in the Nordic country medical context. Perhaps particular for the Nordic countries, which combine medical specialty expertise and practice areas for perioperative medicine, intensive care medicine, pain medicine, and emergency (critical) prehospital care, this document describes current goals in susbpecialty education for the Nordic practice tradition for critical emergency medicine.

## 1 Introduction

**FIGURE 1:**
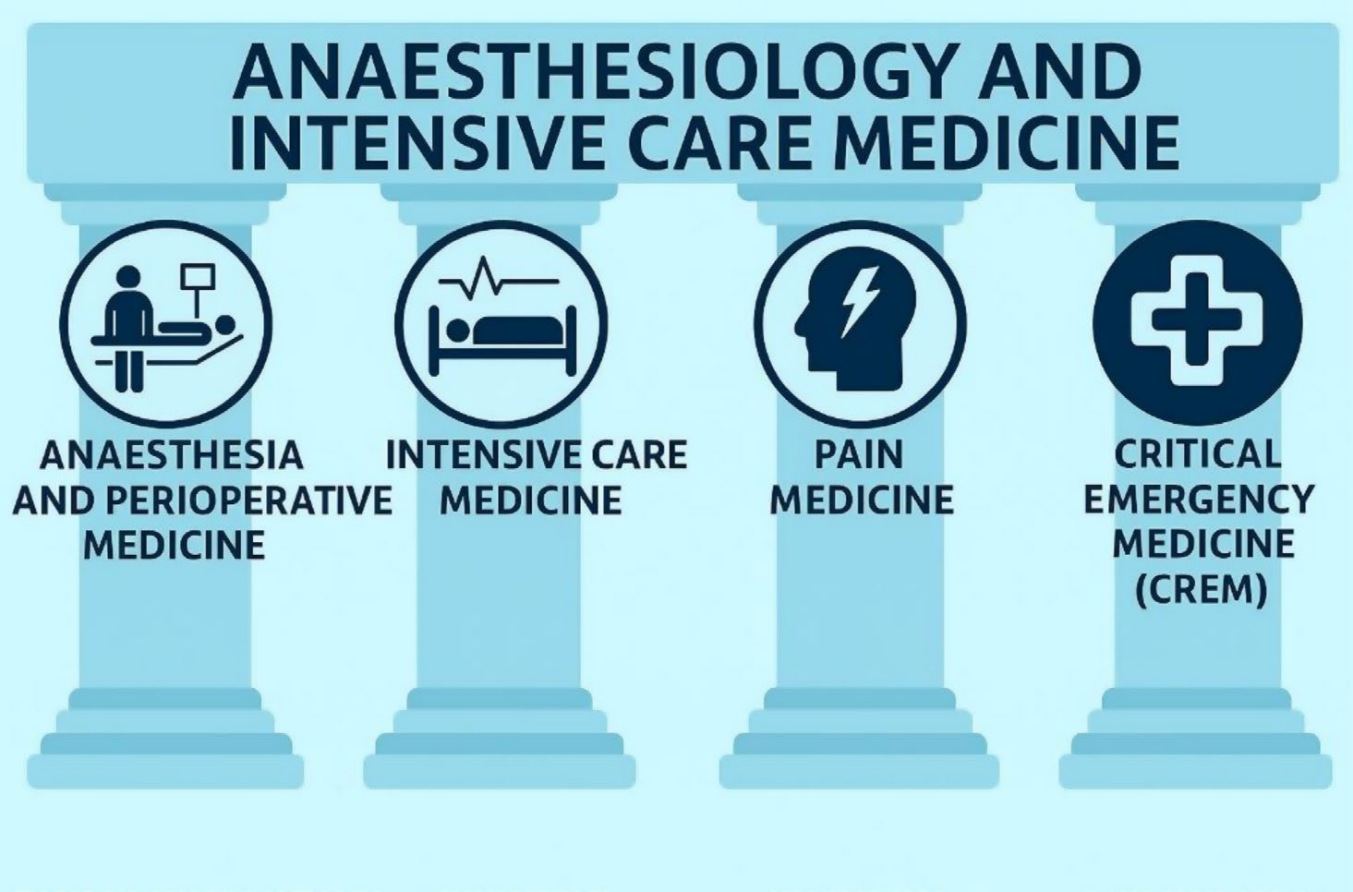
The four pillars of the speciality anesthesiology and intensive care medicine in Scandinavia [1, 2].

Anesthesiology and intensive care medicine is a wide and versatile medical specialty. The umbrella organization for Nordic anesthesiologists, the Scandinavian Society of Anesthesiology and Intensive Care Medicine (SSAI) has defined this specialty as consisting of four pillars: anesthesia and perioperative medicine, intensive care medicine, pain medicine, and critical emergency medicine (CrEM) [1] (Figure 1). An essential part of anesthesia, perioperative, and intensive care medicine is to maintain the vital functions of the patient [3]. For this reason, anesthesiologists are involved in the care of critically ill or injured patients also outside the operation theaters [4]. The importance of mastering the care of an emergency patient is recognized also by the section and board of anesthesiology of the European Union of Medical Specialists (EUMS/UEMS) and they recommended emergency medicine training should be included in the core curriculum in Anesthesiology specialty [4].

Prehospital emergency medicine has been characterized as a physician‐delivered component of prehospital care that enables advanced‐level interventions and initiation of critical care before the arrival at the emergency department [5]. The Scandinavian anesthesiologists have an established role in prehospital critical care and a majority of the physicians in physician‐staffed EMS units in Scandinavian countries are specialists in anesthesiology and intensive care medicine [6, 7]. According to the SSAI's description, CrEM contains immediate life support, and resuscitation actions regardless of location, both inside and outside the hospital setting [1].

Due to the extensive nature of anesthesiology and intensive care medicine as a specialty, SSAI provides several post‐specialist educational programs aiming at different sub‐specialties. Since 2009, CrEM has been one of these educational programs and it has its own curriculum for two‐year advanced training [8]. Although nomenclature is fairly similar, it is important to distinguish CrEM from the primary medical specialty of emergency medicine. CrEM is a relatively new sub‐specialty and up until now, it has lacked a comprehensive definition. The aim of this study was to establish a consensus‐based definition of CrEM and delineate its core components, competencies, and operational domains. The definition is also necessary for the development of both the sub‐specialty itself and the content of its educational program.

## 2 Materials and Methods

### 2.1 Study Design

As with the Oracle in Delphi, the Delphi study process is a way of getting an expert opinion [9] by analysing questions and answers in fields where objective information is hard to reach [10]. The collective opinion of the Scandinavian experts was conducted as a modified Delphi [11]. Participants of the SSAI CrEM program were instructed to write up to two pages in a free assay on what CrEM is. The steering committee generated statements for the Delphi rounds based on the free essays of the participants. The statements were used in two iterative rounds and a final third round with an external validation.

### 2.2 Steering Committee

A steering committee was installed to manage all steps of the study. The steering committee consisted of seven experienced consultants in anesthesiology and intensive care medicine with a sub‐specialization in CrEM from all the Scandinavian countries (Finland, Sweden, Norway, Denmark, and Iceland). The formal connection between the steering committee and the experts was that the experts were participants in the SSAI CrEM course organized by the steering committee. The majority of the steering committee members also contributed to the writing of this article. Given the limited number of anesthesiologists and intensive care physicians in Scandinavia, some personal connections existed between certain participants and members of the steering committee. The steering committee members were not allowed to participate as experts in any of the Delphi rounds of the study.

### 2.3 Selection of Experts

All participants accepted to the 5th SSAI CrEM program were invited to participate in the Delphi study as experts. Participants of the SSAI CrEM program were orally informed during the first residential course about the Delphi study; for each Delphi round the first question was if they would agree to participate in the Delphi study. 21 of the 24 participants “opted in” for this study (Table 1). Admission to this program required documented experience in CrEM, prehospital care, research, leadership, teaching, as well as a motivational letter.

1. Experience in CrEM
2. Prehospital experience
3. Research experience
4. Planned preparation for the program (research and clinical)
5. Leadership experience
6. Other academical or teaching experience
7. Motivational letter

**TABLE 1:** Demographic data of participants.

	First Delphi round	Second Delphi round	Third Delphi round (external validation)
All	21	21	23
Men	17	17	18
Women	4	4	5
Age, years median (IQR)	46 (8)	46 (8)	50 (%)
Years as a specialist, median (IQR)	6 (5)	6 (5)	15 (5)
Years in prehospital work, median (IQR)	8 (7)	8 (7)	18 (8)
Ph D, number (percent)	5 (24%)	5 (24%)	10 (43%)
Articles, median (IQR)	3 (5)	3 (5)	9 (28)

### 2.4 External Validation

For the external validation phase, previous participants from previous SSAI CrEM programs were invited by email. Each invitation included background information outlining the purpose of the study, the significance of participation, and a link to the survey questionnaire for the third round of the Delphi process (external validation). To be considered an external validator, individuals were required to have completed the program (i.e., been awarded the diploma) and provided consent to be included on the alumni list. This list comprised 33 individuals, of whom 23 participated as external validators.

### 2.5 Formulating Statements

The steering committee used all the essays written by the SSAI CrEM course participants (*n* = 24) to identify statements that were then included in the Delphi study. For statements that did not reach 90% consensus, the steering committee reviewed and revised them until a version was agreed upon by all steering committee members. Experts' comments explaining the reasons for disagreement were carefully considered and used to guide the modifications.

### 2.6 Delphi First Round

In the first Delphi round, the experts were asked to provide demographic information, including sex, age, additional credentials, and country of residence, presented in Table 1. After completing this section, they were presented with a summary of the essays, distilled into 44 statements. Participants were asked to either agree or disagree with each statement. If they disagreed, they were prompted to provide a rationale in a free‐text format. After the statements, the experts had the opportunity to add statements or provide comments in a free‐text format.

Following the first Delphi round, the steering committee established a consensus threshold of > 90%, based on the high overall consensus for the statements. Six statements did not meet this criterion and were subsequently revised by the steering committee.

### 2.7 Second Delphi Round

In the second Delphi round, the revised statements were re‐evaluated to assess consensus. A single consensus threshold of > 90% was maintained, given the aim of defining a subspecialty. The six revised statements from the first round were included, along with two additional statements derived from the free‐text comments provided in the first round. After the statements, the experts had the opportunity to add statements or provide comments in a free‐text format. No further rounds were conducted to increase consensus among the experts. Statements that did not reach the consensus threshold are presented in Appendix 4.

### 2.8 Third Delphi Round (External Validation)

In the third and final Delphi round, the final set of statements was evaluated for consensus by the external validators. The external validators were not provided with the consensus levels from the previous rounds.

## 3 Analysis

Of the 39 statements presented, only two failed to reach the predefined consensus threshold of > 90% and are shown in Appendix 6. The remaining 37 statements were organized into 6 thematic categories. Thematic categorization was used to sort final consensus statements into coherent domains reflecting different aspects of CrEM practice. This process was conducted after the final Delphi round and aimed to enhance the interpretability and applicability of the results. A deductive approach was applied. Statements were reviewed and grouped into thematic domains.

### 3.1 Data Collection Methods

Data were systematically collected through the first two Delphi rounds using the Moodle system for electronically handling the administration of students in a university setting (Moodle 4.1, Turku University Finland). The external validation data collection was made by microsoft forms and delivered by email. Two reminders were sent to include as many of the answers as possible.

## 4 Results

### 4.1 Response Rate

The response rate was 88% (21/24) in the first Delphi round, 100% (21/21) in the second round and 70% (23/33) in the final validation round.

### 4.2 The First Delphi Round

**FIGURE 2:**
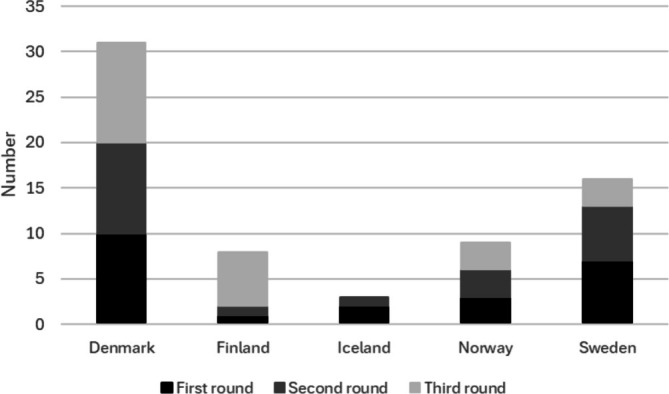
Distribution between nationalities during the three Delphi rounds.

For the first round, 21 participants in the SSAI CrEM program participated: 4 women and 17 men. Demographics are shown in Table 1 and the distribution between nationalities is in Figure 2, One should note that Iceland did not have any alumni. There were 44 statements; 6 statements did not reach a 90% consensus and were revised for the second round. All statements are in Appendix 1. Consensus in percent for all statements is in Appendix 2. Statements with no consensus are shown in Appendix 3.

### 4.3 The Second Delphi Round

Demographics in the second round was the same as in the first Delphi round shown in Table 1 and in Figure 2. In the second round the revised statements F10, F12, F22, F28, F29, and F34 were included as well as two new statements based on free comments from the first Delphi round. Only one statement reached > 90% consensus. Statements with no consensus and examples of comments are shown in Appendix 4. Consensus in percent for the statements is in Appendix 5. All statements are in Appendix 1.

### 4.4 The Third Delphi Round (External Validation)

In the third Delphi round, 23 external validators participated, 5 women and 18 men. Demographics are shown in Table 1 and distribution between nationalities is in Figure 2.

**FIGURE 3:**
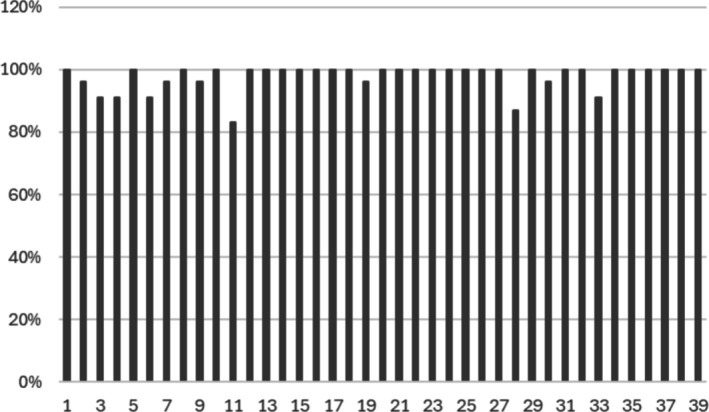
Consensus in percent for all statements (1–39) in the third Delphi round (external validation).

In the third Delphi round (external validation) the statements with consensus > 90% from the first and second rounds were included, in total 39 statements. The two statements with no consensus and examples of comments are shown in Appendix 6. Consensus in percent for all statements in the third Delphi round (external validation) is in Figure 3. All the statements are in Appendix 1.

The statements with a consensus > 90% were grouped into the six thematic categories: core function and scope, competence and training, work environment and challenges, interdisciplinary and teamwork approach, ethical and decision‐making responsibilities, need for research and continuous development (Table 2).

**TABLE 2:** Final statements after the third Delphi round (external validation) with > 90% consensus.

Number	Previous number	Statement	Number (%)
Core function and scope			
1	F1	CrEM overlaps many other medical fields within anesthesiology and intensive care.	21 (100%)
2	F2	CrEM is a fundamental part of the medical work for a Scandinavian anesthesiologist	20 (96%)
3	F3	CrEM requires the knowledge of a physician	19 (91%)
4	F4	CrEM is a sub‐specialty that consists of resuscitation, immediate life support, and prehospital medicine	19 (91%)
6	F6	CrEM focus on immediate life‐saving interventions or other therapies before reaching a specific diagnosis	19 (91%)
7	F7	CrEM includes stabilizing disturbed vital functions in order to enable accurate diagnostics and definitive care	20 (96%)
8	F8	CrEM includes bringing and giving the necessary treatment to the acute critical ill patient, and to stabilize the patient and prevent further deterioration	21 (100%)
9	F9	In addition to managing the patients acute medical condition, CrEM also involves dressing any underlying commodities or chronic conditions, which may require careful management to prevent complications	20 (96%)
12	F14	CrEM includes dealing with medical illness and injuries	21 (100%)
13	F15	CrEM deals with time critical situations (immediate)	21 (100%)
14		CrEM includes being skilled in evaluating the urgency, the nature and the degree of threat to the patient	21 (100%)
16	F18	CrEM changes the outcome for the patient	21 (100%)
17	F19	CrEM includes the preparedness to master difficult and rare medical interventions in challenging environments	21 (100%)
18	F20	One aspect of CrEM is to keep preparedness for major incidents	21 (100%)
20	F23	CrEM includes a high level of knowledge in triage	21 (100%)
27	F32	CrEM requires continuous reevaluation when it comes to diagnosis and treatment	21 (100%)
33	F39	CrEM is management and leadership in mass cass situations, major damage sites on strategic, operational, and tactical level	19 (91%)
34	F40	CrEM is insight in organizational needs, knowledge in planning for new setups for the acute ill patient	21 (100%)
Competence and training			
5	F5	CrEM physicians are highly trained professionals who possess advanced knowledge and skills in managing complex medical conditions	21 (100%)
23	F26	There is a need for a specific education and training in CrEM	21 (100%)
24	F27	To be a good provider of CrEM it is important keeping up your competencies in skills rarely used.	21 (100%)
Work environment and ehallenges			
15	F17	CrEM includes transporting patients to suitable treatment facilities and supporting vital functions during a transport	21 (100%)
19	F21	CrEM practitioners utilize various medical technologies, including advanced imaging, monitoring devices, and life support systems, to effectively manage critical emergencies.	20 (96%)
21	F24	CrEM is not confined to a specific location	21 (100%)
22	F25	CrEM includes working in adverse environments	21 (100%)
26	F31	CrEM is making medical decisions based on limited information	21 (100%)
29	F35	CrEM involves making critical decisions under time constraints, prioritizing interventions based on the severity and urgency of each patient's condition	21 (100%)
31	F37	Beyond the initial resuscitation and stabilization, CrEM professionals may also play a role in coordinating ongoing care, including transferring patients to specialized units or collaborating with intensive care teams	21 (100%)
32	F38	CrEM involves the correct referral of the patient to the relevant treatment	21 (100%)
Interdisciplinary and teamwork approach			
35	F41	CrEM includes respect for a multidisciplinary approach to the patient care, emphasizing the team approach and communication skills	21 (100%)
36	F42	CrEM practitioners has an ability to cooperate with all medical specialties and personnel of different background	21 (100%)
37	F43	CrEM practitioners have the versatility to be able to work in shifting diverse teams as leaders and as followers	21 (100%)
38	F44	CrEM involves being and excellent team leader both enabling the team to heighten the performance and encouraging the mutual care of all the members in difficult situations	21 (100%)
Ethical and decision‐making responsibilities			
11	F11	CrEM includes the ability to know when NOT to treat	21 (100%)
30	F36	CrEM involves the management of complex ethical and legal issues	20 (96%)
39	S7	In CrEM you should strive for the best possible treatment given the situation, resources and urgency and may have to agree to refrain from interventions one would have done with more resources at a different location	21 (100%)
Need for research and continuous development			
25	F30	There is a need for research in the treatment and management in the prehospital setup	21 (100%)

## 5 Discussion

**FIGURE 4:**
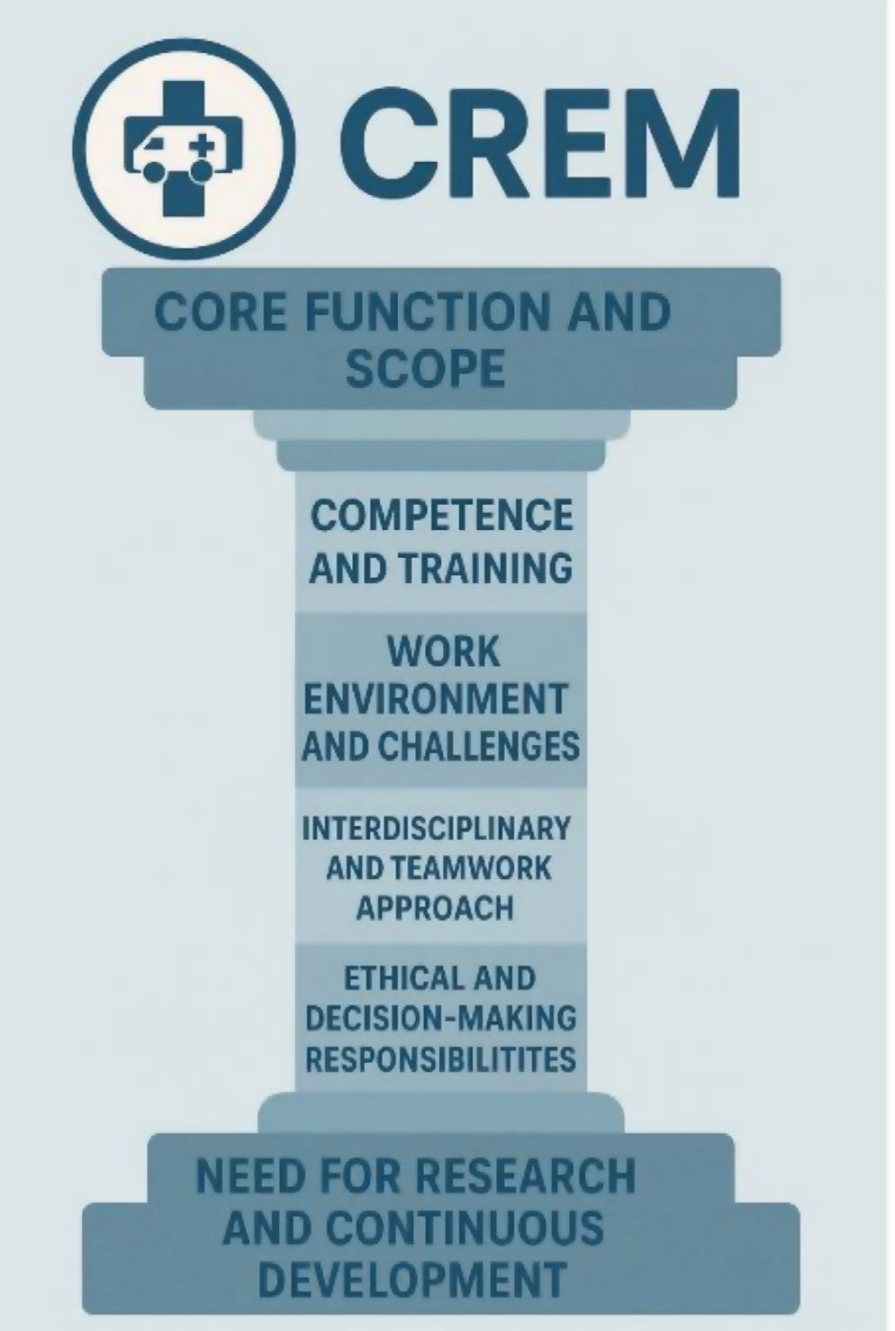
The CrEM pillar with the six thematic domains.

This study is the first to attempt to define and chisel the pillar of the subspecialty of CrEM. CrEM has previously been described as one of the four pillars of the Scandinavian specialty Anesthesia and intensive care medicine [1] (Figure 1). The conceptualization of CrEM may be illustrated as a structural pillar, symbolizing the foundational and integrative nature of the subspecialty (Figure 4). The CrEM pillar, as the other three rests on the base of research and continuous development, although this pillar is not yet as unearthed as the other three (perioperative, intensive care and pain medicine). On the top of the pillar lies the core function and scope of CrEM. Framing the core of the pillar are four essential and interdependent domains that characterize both the operational and professional framework of CrEM: competence and training, work environment and challenges, interdisciplinary and teamwork approach, and ethical and decision‐making responsibilities. Establishing a widely accepted definition is essential for the advancement of CrEM.

### 5.1 Core Function and Scope

Consensus from this study is that CrEM is a subspecialty within anesthesiology and intensive care medicine that constitutes a fundamental part of the medical work for Scandinavian anesthesiologists. CrEM involves stabilizing disrupted vital functions to enable precise diagnostics and definitive care, as well as delivering necessary treatment to critically ill patients to prevent further deterioration. CrEM overlaps with several other medical disciplines and focuses on immediate life‐saving interventions and therapies before a definitive diagnosis is established. It encompasses resuscitation, prehospital medicine, and acute emergency interventions, where physician‐level expertise is essential to ensure accurate assessment and appropriate treatment. Prehospital physicians have been shown to positively influence patient outcomes not only in Scandinavia but also in other parts of the world [12, 13].

According to our findings, a key aspect of CrEM is managing time‐critical situations, requiring the ability to rapidly evaluate the urgency, nature, and degree of threat to the patient. CrEM significantly impacts patient outcomes by enabling rapid and effective interventions that can be crucial for survival and recovery. Time is a crucial factor in several different interventions; for example, the time to intervention can impact a patient's survival [14], and the time to blood transfusion is critical for a bleeding patient [15]. Together, these aspects create a dynamic where speed and time significantly influence the CrEM practitioner.

The consensus was that CrEM demands expertise in prehospital and hospital‐based emergency systems, including triage, organization, and leadership in mass casualty incidents and large‐scale emergencies at strategic, operational, and tactical levels. In addition to addressing acute medical conditions, CrEM also involves managing underlying comorbidities or chronic conditions to minimize complications. This dynamic field necessitates continuous reassessment of diagnosis and treatment, as well as preparedness to perform rare and complex medical interventions in challenging environments.

We also found that the field requires an in‐depth understanding of organizational needs and planning for new setups in the care of critically ill patients. By combining medical expertise, leadership, and operational readiness, CrEM plays a pivotal role in managing critically ill and injured patients, highlighting its importance as a key part of modern anesthesiology and intensive care medicine.

### 5.2 Competence and Training

The consensus was that due to the demanding nature of CrEM, there is a clear need for specialized education and structured training programs to ensure proficiency in both fundamental and advanced life‐saving interventions. As in other specialties within anesthesia and intensive care, there is a clear need for specialized education and training. The SSAI offers a range of postgraduate programs across various subspecialties. In certain fields, such as pediatric anesthesia, it is widely expected that physicians complete a dedicated training program to be recognized as senior practitioners. The duration and structure of these programs vary, with a 12‐month fellowship being a common format in pediatric anesthesia [16].

Simulation and practice enhance confidence and prepare personnel for rare events [17, 18]. We also found that maintaining competence in rarely used but critical skills is essential for effective CrEM practice. Continuous training and skill retention strategies are crucial to ensure that CrEM providers can deliver high‐quality care in unpredictable and high‐stakes environments. For example, the outcomes of prehospital anesthesia seem to be associated with the frequency of procedure performance, with lower mortality rates, reduced on‐scene time, and a higher first‐pass success rate for intubation [19, 20].

### 5.3 Work Environment and Challenges

The consensus was that CrEM is a dynamic and location‐independent specialty that encompasses patient stabilization, transport to appropriate treatment facilities, and the continuous support of vital functions during transit. CrEM practitioners operate in diverse and often adverse environments, utilizing advanced medical technologies such as imaging, monitoring devices, and life support systems to manage critical emergencies effectively. Although the prehospital environment is not the only place where CrEM is performed it is worth noting that the prehospital environment presents challenges for physicians [21].

We also found that decision‐making in CrEM is frequently based on limited information and must be executed under significant time constraints, requiring rapid prioritization of interventions based on severity and urgency. Beyond initial resuscitation and stabilization, CrEM professionals play a crucial role in coordinating ongoing patient care, ensuring appropriate referrals, and facilitating seamless transitions to specialized units or intensive care teams. Their ability to make high‐stakes decisions in challenging conditions is essential for optimizing patient outcomes; one example is airway management in the prehospital environment [22].

### 5.4 Interdisciplinary and Teamwork Approach

We found that CrEM is fundamentally based on a multidisciplinary approach, emphasizing teamwork and effective communication in high‐stakes environments. There is a clear link between teamwork and patient safety [23].

The consensus was that CrEM practitioners collaborate seamlessly with medical professionals across various specialties and backgrounds, demonstrating both leadership and adaptability in diverse and shifting teams. Their ability to function as both leaders and team members ensures the optimal delivery of patient care in dynamic settings. Effective CrEM leadership involves not only guiding the team to enhance performance; it also includes fostering a supportive environment that prioritizes mutual care and resilience (the ability of individuals and teams to recover, adapt, and continue functioning effectively), particularly in challenging and high‐pressure situations. Strong teamwork and communication skills are essential components of CrEM, directly influencing patient outcomes and overall team efficiency [3].

### 5.5 Ethical and Decision‐Making Responsibilities

We found that CrEM requires not only advanced clinical skills but also the ability to recognize when not to intervene. CrEM practitioners must navigate complex ethical and legal considerations while making high‐stakes decisions under resource‐limited and time‐sensitive conditions. Several non‐medical factors influence decision‐making in the prehospital setting, including patient‐related factors, bystander influences, the personal circumstances of healthcare providers, and external factors [24].

Consensus is that striving for the best possible treatment within the constraints of the situation, they must balance urgency, available resources, and patient prognosis. In some cases, this means refraining from interventions that might have been feasible in a different setting with more resources. Ethical judgment, prioritization, and adaptability are therefore essential components of CrEM, ensuring both effective patient care and responsible medical decision‐making.

Decision‐making in out‐of‐hospital cardiac arrest is influenced by expectations regarding the patient's prognosis, the physician's values and beliefs, and the ethical dilemmas encountered during the decision‐making process [25].

### 5.6 Need for Research and Continuous Development

We found that there is a need for CrEM research in the prehospital setting. As prehospital care plays a vital role in patient outcomes, evidence‐based strategies are essential to optimize interventions, improve decision‐making, and enhance resource utilization in these time‐sensitive environments. Research in this field should address best practices for stabilization, triage, transport logistics, and the integration of new medical technologies to ensure the highest standard of care before hospital admission. Advancing knowledge in prehospital medicine will contribute to more effective emergency care systems and better overall patient outcomes.

## 6 Limitations

The statements used in this study were based on essays written by the participants during their training in the SSAI CrEM program, which may have shaped the scope and framing of the statements. This reliance on participant‐generated material could introduce biases related to the curriculum. To ensure that the participants' perspectives did not have an excessive influence, the steering committee adjusted the statements. In each round, there was also an opportunity to suggest additions and share personal opinions about CrEM.

As CrEM lacks a formally or professionally established definition, whether through scientific consensus, specialty board recognition, or standardized curricula, it was not feasible to define the field solely through existing literature. In this context, building on the perspectives of experienced practitioners was considered a valid and appropriate approach to explore and articulate the boundaries of CrEM. The two initial rounds were conducted with participants who were still in the process of specializing to become experts, which may influence the depth of their insights compared to fully trained specialists. The final validation round was conducted with previous participants who have since become experts in the field. This ensures that the final statements are reviewed by individuals with specialized knowledge.

As with all Delphi studies, the consensus‐driven nature of the methodology may limit the inclusion of minority opinions or novel perspectives that do not align with the majority view. A declining response rate is a common challenge in the Delphi method; however, in this study, the response rate remained high across all three rounds. The relatively small size of the expert panel in our study means that the influence of a single respondent on the overall percentage could be substantial. As seen in Appendices 4 and 6, many items approached the 90% consensus threshold, and the inclusion or exclusion of one respondent could have shifted results significantly. This inherent sensitivity should be taken into account when interpreting the strength of consensus for borderline items. While consensus thresholds of 70%–75% are commonly used in Delphi studies, we chose a more conservative threshold of 90% to ensure that only statements with the strongest possible agreement were included. A lower threshold would have resulted in consensus on nearly all statements, thereby limiting the ability to identify which elements truly reflect core shared understandings within the field.

While CrEM is a key focus for Scandinavian anesthesiologists and intensive care physicians, many other healthcare professionals also encounter these patients and require relevant expertise. The need for knowledge and development among anesthesia and intensive care physicians does not preclude the importance of other medical professionals, including physicians, nurses, and paramedics, acquiring competence in this field.

## 7 Conclusion

CrEM is now defined as a subspecialty within anesthesiology and intensive care medicine, focusing on rapid, life‐saving interventions across diverse and often unpredictable environments. This study identifies six core domains, ranging from clinical scope and competence to ethical responsibility and the need for continued research, that collectively shape the foundation of CrEM. The results emphasize the importance of structured training, interdisciplinary collaboration, and the ability to make high‐stakes decisions under pressure. As a dynamic and evolving field, CrEM requires continuous professional development and increased research efforts, particularly within prehospital care, to ensure the highest standards of patient outcomes and system resilience.

## Author Contributions

D.B. designed the study. D.B., M.T., M.P., T.L., N.K., and R.L. participated as the steering committee and revised the statements. D.B. did the statistical calculations. D.B., M.T., and R.L. wrote the manuscript. All authors have read and agreed to the final version of the manuscript.

## Ethics Statement

This study was performed in accordance with the ethical standards as laid down in the 1964 declaration of Helsinki and its later amendments. Ethical approval was not necessary for this study, as it did not include sensitive personal information or data subject to ethical review according to the Swedish ethical review authority. This decision is in line with the regulations set forth by the Swedish Research Council (2017) https://etikprovningsmyndigheten.se/en/about‐the‐authority/.

## Consent

Participants in the first two Delphi rounds were provided with verbal information regarding the purpose of the study and were given the opportunity to ask questions. For the third round, participants received written information outlining the study's objectives. In each round, the first item in the questionnaire asked participants to confirm their informed consent to take part in the Delphi process. Accordingly, informed consent was obtained at each stage of participation.

## Conflicts of Interest

The authors declare no conflicts of interest.

## Data Availability

The data that support the findings of this study are available from the corresponding author upon reasonable request.

## Appendix 1 First, Second, and Third Delphi Rounds With Percentages for All Statements. Statements Without Consensus (< 90%) Are Highlighted in Gray

**Table:**

First Delphi round		
Number	Statement	%
F1	CrEM overlaps many other medical fields within anesthesiology and intensive care	100%
F2	CrEM is a fundamental part of the medical work for a Scandinavian anesthesiologist	100%
F3	CrEM requires the knowledge of physician	95%
F4	A sub‐specialty that consists of resuscitation, immediate life support and prehospital medicine	90%
F5	CrEM physicians are highly trained professionals who possess advanced knowledge and skills in managing complex medical conditions	90%
F6	CrEM focus on immediate life‐saving interventions or other therapies before reaching a specific diagnosis	95%
F7	CrEM includes stabilizing disturbed vital functions in order to enable accurate diagnostics and definitive care	100%
F8	CrEM includes bringing and giving the necessary treatment to the acute critical ill patient, and to stabilize the patient and prevent further deterioration	100%
F9	In addition to managing the patients acute medical condition, CrEM also involves dressing any underlying commodities or chronic conditions, which may require careful management to prevent complications	90%
F10	CrEM includes an enhanced knowledge in disease and parthenogenesis and an enhanced knowledge in the treatment	76%
F11	CrEM includes the ability to know when NOT to treat	100%
F12	CrEM includes advanced knowledge in pain management	86%
F13	CrEM is a cross‐sectional subspecialty that borderlines many other specialties	90%
F14	CrEM includes dealing with medical illness and injuries	90%
F15	CrEM deals with time critical situations (immediate).	100%
F16	CrEM includes being skilled in evaluating the urgency, the nature and the degree of threat to the patient	95%
F17	CrEM includes transporting patients to suitable treatment facilities and supporting vital functions during a transport	100%
F18	CrEM changes the outcome for the patient	90%
F19	CrEM includes the preparedness to master difficult and rare medical interventions in challenging environments	95%
F20	One aspect of CrEM is to keep preparedness for major incidents	95%
F21	CrEM practitioners utilize various medical technologies, including advanced imaging, monitoring devices, and life support systems, to effectively manage critical emergencies.	95%
F22	CrEM includes healthcare resilience	77%
F23	CrEM includes a high level of knowledge in triage	100%
F24	CrEM is not confined to a specific location	100%
F25	CrEM includes working in adverse environments	100%
F26	There is a need for a specific education and training in CrEM	95%
F27	To be a good provider of CrEM it is important keeping up your competencies in skills rarely used.	100%
F28	Working with CrEM you should have a sense of excellence – especially on the ‘easy stuff’, which should be carried out excellent every time	86%
F29	There is a need for research in CrEM, especially compared to other medical fields	81%
F30	There is a need for research in the treatment and management in the prehospital setup	100%
F31	CrEM is making medical decisions based on limited information	95%
F32	CrEM requires continuous reevaluation when it comes to diagnosis and treatment	95%
F33	CrEM leans more toward treating symptoms than diagnoses	90%
F34	CrEM is making quick medical decisions under a lot of pressure and handling patients usually without a backup	81%
F35	CrEM involves making critical decisions under time constraints, prioritizing interventions based on the severity and urgency of each patient's condition	100%
F36	CrEM involves the management of complex ethical and legal issues	90%
F37	Beyond the initial resuscitation and stabilization, CrEM professionals may also play a role in coordinating ongoing care, including transferring patients to specialized units or collaborating with intensive care teams	100%
F38	CrEM involves the correct referral of the patient to the relevant treatment	100%
F39	CrEM is managemant and leadership in mass cass situations, major damage sites on strategic, operational and tactical level	90%
F40	CrEM is insight in organizational needs, knowledge in planning for new setups for the acute ill patient	100%
F41	CrEM includes respect for a multidisciplinary approach to the patient care, emphasizing the team approach and communication skills	95%
F42	CrEM practitioners has an ability to cooperate with all medical specialties and personnel of different background	90%
F43	CrEM practitioners have the versatility to be able to work in shifting diverse teams as leaders and as followers	95%
F44	CrEM involves being and excellent team leader both enabling the team to heighten the performance and encouraging the mutual care of all the members in difficult situations	100%

Second Delphi round			
Number	Previous number	Statement	%
S1	F10	CrEM physicians need a broad base of knowledge of physiology and pathophysiology	76%
S2	F12	CrEM includes advanced knowledge in acute pain management	86%
S3	F22	Because CrEM often requires working in challenging circumstances, health care worker resilience is important for the CrEM specialist	76%
S4	F28	In CrEM the basic tasks should consistently be managed with proficiency	86%
S5	F29	Given the relative shortage of research in the area of CrEM, there is need for an intensified effort	81%
S6	F34	CrEM is making quick medical decisions under a lot of pressure and handling patients with limited backup	81%
S7		In CrEM you should strive for the best possible treatment given the situation, resources and urgency and may have to agree to refrain from interventions one would have done with more resources at a different location.	100%
S8		CrEM is the support of vital functions which is more important than pin‐pointing a precise diagnose	86%

Third Delphi round (external validation)			
Number	Previous number	Statement	%
1	F1	CrEM overlaps many other medical fields within anesthesiology and intensive care.	100%
2	F2	CrEM is a fundamental part of the medical work for a Scandinavian anesthesiologist	96%
3	F3	CrEM requires the knowledge of a physician	91%
4	F4	CrEM is a sub‐specialty that consists of resuscitation, immediate life support and prehospital medicine	91%
5	F5	CrEM physicians are highly trained professionals who possess advanced knowledge and skills in managing complex medical conditions	100%
6	F6	CrEM focus on immediate life‐saving interventions or other therapies before reaching a specific diagnosis	91%
7	F7	CrEM includes stabilizing disturbed vital functions in order to enable accurate diagnostics and definitive care	96%
8	F8	CrEM includes bringing and giving the necessary treatment to the acute critical ill patient, and to stabilize the patient and prevent further deterioration	100%
9	F9	In addition to managing the patients acute medical condition, CrEM also involves dressing any underlying commodities or chronic conditions, which may require careful management to prevent complications	96%
10	F11	CrEM includes the ability to know when NOT to treat	100%
11	F13	CrEM is a cross‐sectional subspecialty that borderlines many other specialties	83%
12	F14	CrEM includes dealing with medical illness and injuries	100%
13	F15	CrEM deals with time critical situations (immediate)	100%
14		CrEM includes being skilled in evaluating the urgency, the nature and the degree of threat to the patient	100%
15	F17	CrEM includes transporting patients to suitable treatment facilities and supporting vital functions during a transport	100%
16	F18	CrEM changes the outcome for the patient	100%
17	F19	CrEM includes the preparedness to master difficult and rare medical interventions in challenging environments	100%
18	F20	One aspect of CrEM is to keep preparedness for major incidents	100%
19	F21	CrEM practitioners utilize various medical technologies, including advanced imaging, monitoring devices, and life support systems, to effectively manage critical emergencies.	96%
20	F23	CrEM includes a high level of knowledge in triage	100%
21	F24	CrEM is not confined to a specific location	100%
22	F25	CrEM includes working in adverse environments	100%
23	F26	There is a need for a specific education and training in CrEM	100%
24	F27	To be a good provider of CrEM it is important keeping up your competencies in skills rarely used.	100%
25	F30	There is a need for research in the treatment and management in the prehospital setup	100%
26	F31	CrEM is making medical decisions based on limited information	100%
27	F32	CrEM requires continuous reevaluation when it comes to diagnosis and treatment	100%
28	F33	CrEM leans more toward treating symptoms than diagnoses	87%
29	F35	CrEM involves making critical decisions under time constraints, prioritizing interventions based on the severity and urgency of each patient's condition	100%
30	F36	CrEM involves the management of complex ethical and legal issues	96%
31	F37	Beyond the initial resuscitation and stabilization, CrEM professionals may also play a role in coordinating ongoing care, including transferring patients to specialized units or collaborating with intensive care teams	100%
32	F38	CrEM involves the correct referral of the patient to the relevant treatment	100%
33	F39	CrEM is managemant and leadership in mass cass situations, major damage sites on strategic, operational and tactical level	91%
34	F40	CrEM is insight in organizational needs, knowledge in planning for new setups for the acute ill patient	100%
35	F41	CrEM includes respect for a multidisciplinary approach to the patient care, emphasizing the team approach and communication skills	100%
36	F42	CrEM practitioners has an ability to cooperate with all medical specialties and personnel of different background	100%
37	F43	CrEM practitioners have the versatility to be able to work in shifting diverse teams as leaders and as followers	100%
38	F44	CrEM involves being and excellent team leader both enabling the team to heighten the performance and encouraging the mutual care of all the members in difficult situations	100%
39	S7	In CrEM you should strive for the best possible treatment given the situation, resources and urgency and may have to agree to refrain from interventions one would have done with more resources at a different location	100%

## Appendix 3 First Delphi Round Statements With No Consensus (< 90%) and Examples of Comments

**Table:**

First Delphi round statements with no consensus (< 90%)			
Number	Statements	Example of comments	Number (%)
F10	CrEM includes an enhanced knowledge in disease and parthenogenesis and an enhanced knowledge in the treatment	“I'm not sure enhanced knowledge in disease and pathogenesis is necessary in CrEM. Maybe in specific conditions, but not in the general sense the question is asked” “Pathogensis—perhaps not so much. Disease and treatment, yes”	17 (77%)
F12	CrEM includes advanced knowledge in pain management	“The CrEM will involve some pain management but mostly in a rather acute setting.”	18 (86%)
F22	CrEM includes healthcare resilience	“I am not sure what that statement means?” “Health care resilience is not a concept unique to CrEM but would be applicable to all medical fields in the event of disrupture.”	16 (77%)
F28	Working with CrEM you should have a sense of excellence—especially on the “easy stuff”, which should be carried out excellent every time	“To strive for excellency is not a concept unique for CrEM.”	18 (86%)
F29	There is a need for research in CrEM, especially compared to other medical fields	“why…espesically compared to other medical fields” “Maybe reframed: given the relative shortage of research in the area of CrEM, there is need of an intensified effort?”	17 (81%)
F34	CrEM is making quick medical decisions under a lot of pressure and handling patients usually without a backup	“CrEM does not have to be a single‐doctor show. Backup may be available also in CrEM, but then again, it may not.” “I sort of agree but the phrasing does not take into account that practising CrEM can be anywhere. If in a hospital 1) there may be many around you thus you are not alone 2) CrEM may deal with pressure but it somewhat created by yourself and thus varies from person to person and CrEM does not only deal with situations of pressure.”	17 (81%)

## Appendix 4 Second Delphi Round Statements With No Consensus (< 90%) and Examples of Comments

**Table:**

Second Delphi round statements with no consensus (< 90%)				
Number	Previous number	Statements	Example of comments	Number (%)
S1	F10	CrEM physicians need a broad base of knowledge of physiology and pathophysiology	“Basic Knowledge in disease, pathogenesis and treatment must surely exist in every physician, I'm not sure enhanced knowledge in disease and pathogenesis is necessary in CrEM. Maybe in specific conditions, but not in the general sense the question is asked”	16 (77%)
S2	F12	CrEM includes advanced knowledge in acute pain management	“CrEM practitioners will utilize their knowledge of pain management as specialist but I understand advanced pain knowledge as something that creeps outside of the CrEM curriculum. More into an advanced pain curriculum.”	18 (86%)
S3	F22	Because CrEM often requires working in challenging circumstances, health care worker resilience is important for the CrEM specialist	“Health care resilience is not a concept unique to CrEM but would be applicable to all medical fields in the event of disrupture.”	16 (77%)
S4	F28	In CrEM the basic tasks should consistently be managed with proficiency	“CrEM involves the ability to judge when something is good‐enough or might‐not‐become‐better under certain circumstances. This ability can of course be excellent but to always do everything perfect can sometimes be harmful and therefore the opposite of excellent care.”	18 (86%)
S5	F29	Given the relative shortage of research in the area of CrEM, there is need for an intensified effort	“CrEM is indeed in need of research, but just because it isn't studied earlier doesn't make it in more need than other fields. CrEM could use the knowledge of what is good research and leverage that in gaining good and useful knowledge.”	18 (86%)
S6	F34	CrEM is making quick medical decisions under a lot of pressure and handling patients with limited backup	“CrEM does not have to be a single‐doctor show. Backup may be available also in CrEM, but then again, it may not.”	17 (81%)
S8		CrEM is the support of vital functions which is more important than pin‐pointing a precise diagnose	“Not always, sometimes its also straingthforward.”	18 (86%)

## Appendix 6 Third Delphi Round (External Validation) Statements With No Consensus (< 90%) and Examples of Comments

**Table:**

Third Delphi round (external validation) statements with no consensus (< 90%)				
Number	Previous number	Statement	Example of comments	Number (%)
11	F13	CrEM is a cross‐sectional subspecialty that borderlines many other specialties	“The sacndinavian anaesthesthetist is best suited for this” “Advanced resuscitation is performed by a few specialities”	83%
28	F33	CrEM leans more toward treating symptoms than diagnoses	“Not entirely, b/c often times you have enough of a diagnosis to treat the injury/disease rather than its symptom” “Although ABC is mostly treating symptoms, usually the (working) diagnosis is the thing that guides our decisions and actions, for example airway burn injury → early intubation for A, traumatic chest injury → needle thoracostomy for B and so on”	87%
